# Elusive Cardiac Angiosarcoma in a Young Pregnant Female: Rare Presentation With Fatal Outcome

**DOI:** 10.14740/cr402w

**Published:** 2015-06-11

**Authors:** Abdelkarim Waness, Amal A. Batoon, Imran Mirza, Wael Al Mahmeed

**Affiliations:** a Obstetrics and Gynecology Department, Sidra Medical and Research Center, Doha, Qatar; b Department of Medicine, Sheikh Khalifa Medical City, Abu Dhabi, United Arab Emirates; c Department of Pathology, Sheikh Khalifa Medical City, Abu Dhabi, United Arab Emirates; d Department of Cardiology, Sheikh Khalifa Medical City, Abu Dhabi, United Arab Emirates

**Keywords:** Pregnancy, Pericardial effusion, Right atrium angiosarcoma, Echocardiography, Magnetic resonance imaging, Paclitaxel

## Abstract

Heart tumors are rare occurrences. They can present diagnostic challenges and severe complications especially in pregnant women. We report a rare case of angiosarcoma (AS) cordis of the right atrium in a young healthy pregnant female. Her diagnosis remained elusive for some time until development of advanced disease symptomatology. The diagnosis was unfortunately clinched when her tumor grew to be detected by imaging modalities. An emergency cesarean section was performed delivering a healthy baby. The patient was aggressively treated with chemotherapy to no avail. She passed away 2 months after her diagnosis was established. Only few cases of the occurrence of aggressive cardiac AS and human pregnancy are documented. The course of this disastrous combination is usually marked by severe complications, difficult therapeutic options and ultimately fast demise. Physicians need to be more aware of such diagnosis and diligently try to diagnose it as early as possible.

## Introduction

Cardiac tumors are rare occurrence. Their development however can be marred with complex symptomatology and severe complications. Angiosarcoma (AS) is a uniquely aggressive malignant neoplasm of endothelial cells. It can be encountered in various parts of the body including the heart. The discovery of this worrisome cardiac tumor in a pregnant female has been reported only in few documented instances.

## Case Report

**Figure 1:**
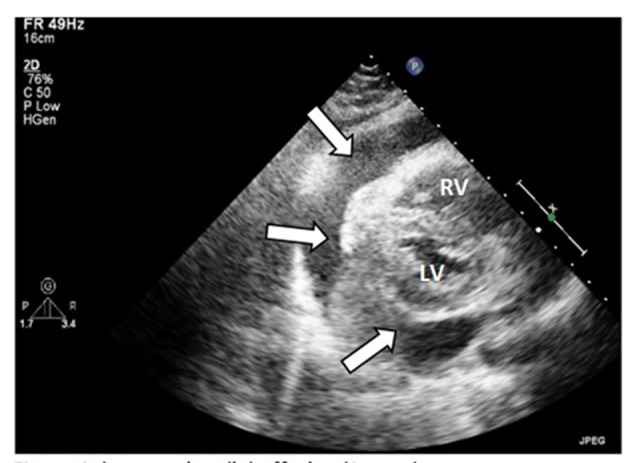
Large pericardial effusion (arrow). RV: right ventricle; LV: left ventricle.

A G2P1 female in her thirties developed dyspnea while 18-week pregnant. She was taking prenatal vitamin, folic acid and occasional bronchodilator inhaler. Her vital signs were: temperature 36.3 °C, pulse 110 bpm, respiratory rate 22 per minute, BP 90/59 mm Hg, and oxygen saturation 97% on room air. On auscultation, she had distant heart sounds with pulsus paradoxus but no murmur or rub. The rest of her exam was normal including jugular venous pressure. Pertinent laboratory findings: hemoglobin 89 g/L, platelets: 223 × 10^9^/L, both anti-nuclear antibodies (ANA) and double-strand DNA antibodies (anti ds-DNA) testing were negative. The rest of her laboratory findings, including coagulation profile and thyroid stimulating hormone (TSH), were unremarkable. EKG showed sinus tachycardia. Transthoracic echocardiogram (TTE) (Fig. 1) demonstrated large pericardial effusion. With impending cardiac tamponade, the patient underwent pericardiocentesis and 800 mL of blood-stained fluid was removed. Cytopathologic and microbiologic examinations of the pericardial fluid showed multiple RBCs without detectable infectious, auto-immune, or malignant causes.

The initial working diagnosis was hemopericardium of unknown etiology. Two weeks post-discharge, a repeat TTE showed only a small pericardial effusion. A third TTE, done 6 weeks afterward, failed to show any apparent fluid collection. The patient unfortunately missed a third follow-up appointment.

**Figure 2:**
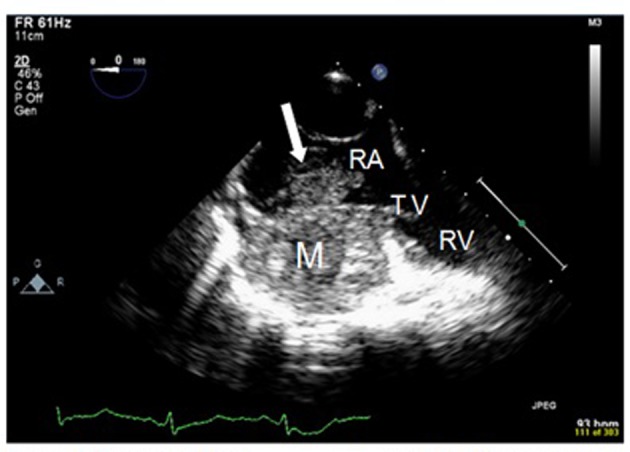
Transesophageal echocardiogram (TOE): transverse view of the right chambers. There is a large firm infiltrating mass (M) in the right atrium (RA), with a propagation (arrow) within the right atrial cavity. RA: right atrium; TV: tricuspide valve; RV: right ventricle.

**Figure 3:**
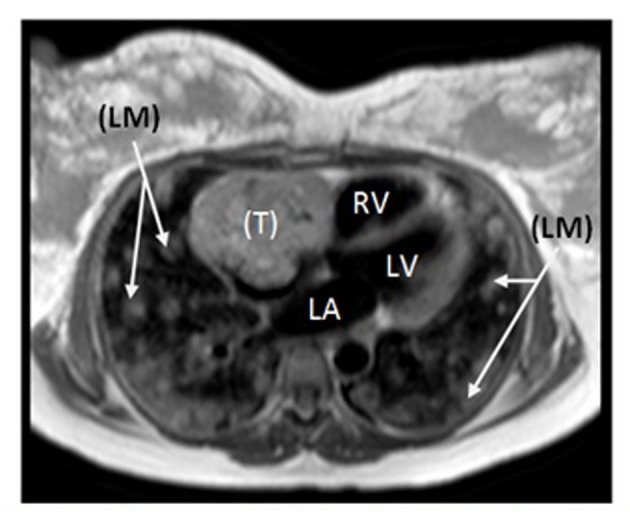
Cardiac MRI revealing large irregular tumor (T) occupying most of the RA, with numerous bilateral lung metastases (LM). RV: right ventricle; LA: left atrium; LV: left ventricle.

**Figure 4:**
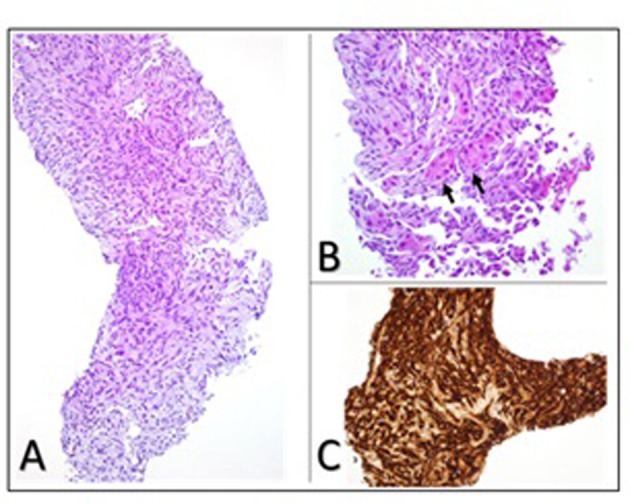
Liver biopsy showing (A) photomicrograph of moderate to poorly differentiated angiosarcoma with dense clumps of plump and spindle shaped, anaplastic cells focally showing slit-like vascular lumens (original magnification, × 200); (B) entrapped hepatocytes (arrows) within the tumor (original magnification, × 400); (C) immunohistochemical staining for the endothelial marker CD34 demonstrating the endothelial origin of the neoplastic cells.

At the 38th week of her pregnancy, the patient developed chest pain. Vital signs were: temperature 37.6 °C, pulse 100 bpm, respiratory rate 19 per minute, BP 113/85 mm Hg, and oxygen saturation 98% on room air. Few crackles were detected on auscultation. Her abdomen was distended (appropriate for pregnancy) while the rest of the exam, including skin and lymph nodes, was negative. Laboratory tests included: hemoglobin 110 g/L, platelets 324 × 10^9^/L, and troponin 0.8 ng/mL. Electrocardiogram showed diffuse ST segment depression with T wave inversion. Trans-thoracic echocardiogram showed a fragile echo-dense mobile structure seen in a dilated right atrium (RA). A transesophageal echocardiography (TOE) confirmed the presence of a large right atrial mass (Fig. 2). Differential diagnoses shifted to possible RA thrombus or RA tumor. The patient underwent an emergency cesarean section delivering a healthy boy. Close uterine and placental inspections were unremarkable. Further evaluation, with computed tomography and magnetic resonance imaging (MRI) of the chest and abdomen, showed not only the presence of the right atrial mass but also multiple nodules scattered in both lungs and liver (Fig. 3). The patient underwent a biopsy of one hepatic lesion that showed moderate to poorly differentiated AS (Fig. 4).

The final diagnosis was metastatic cardiac AS to the lungs and liver. Chemotherapy with paclitaxel was initiated. Six weeks post-partum, a repeat TTE was done: it showed no change in the cardiac tumor size and no detectable pericardial effusion. Despite aggressive therapy, the patient succumbed to death 3 months after establishing her diagnosis.

## Discussion

ASs are rare aggressive malignant tumors emanating from blood vessels’ endothelial cells. Genetic and congenital factors seem to play a role in the genesis of AS [1, 2]. Radiotherapy and vinyl chloride are also believed to contribute to their development [3, 4]. Immunohistological studies have identified factors such as protein kinase p-AKT and the over-expression of vascular endothelial growth factors in the pathogenesis of these tumors [5].

The prevalence of primary cardiac tumors is estimated at 0.01% in post-portem studies [6]. The spectrum of primary cardiac tumors is dominated by benign myxomas. It is reported that 25% of all cardiac tumors are highly aggressive ASs [7]. While myxomas have predilection for the left atrium (LA), AS seem to favor the RA. In a large series of 242 heart tumor cases, Yu and colleagues confirmed this predilection: four out of seven AS (57%) cases originated in the RA [8]. Another 10-year single center experience of 24 cardiac sarcomas pointed to this predilection: 10 out of 24 (41.6%) cases were AS, and all of them (100%) originated in the RA [9]. The mean age for AS in this series was 42.2 years. The occurrence of cardiac AS in younger population is documented by Hamidi and colleagues [10]. There is no apparent gender predilection for these tumors however.

Coincidental occurrence of primary AS cordis with pregnancy is extremely unusual. Only few cases, from 1969 to 2012, were reported in the medical literature [11-15].

Our unfortunate patient had her tumor also originating in the RA. She presented a challenging diagnostic dilemma from the beginning. She had two different clinical and echocardiographic presentations. Initially, she developed a picture of impending cardiac tamponade that was relieved by pericardiocentesis of blood-stained fluid. Two subsequent TTEs gave the false impression of a “possibly benign pericardial effusion”. It took only four short months for this patient’s tumor to herald its aggressive presence in the heart, lungs and liver.

During pregnancy, the placenta secretes a VEGF homolog called the placenta growth factors (PlGFs). PlGF-1 acts as a natural antagonist of VEGF when both factors are simultaneously synthesized in murine fibrosarcoma cells. The exact role(s) of PlGF in cancer growth and dissemination remain to be fully understood however [16].

As illustrated in this case, diagnosing primary AS cordis at an early stage can be very challenging [17]. These tumors usually have late, dramatic and devastating presentation with a variety of constitutional symptoms such as cardiac chamber flow obstruction, valvular dysfunction, cardiac arrhythmias, pericardial effusion, and embolic phenomena [18]. Complications might include: syncope [19], superior vena cava syndrome [20], spontaneous right atrial rupture resulting in cardiac tamponade [21], and even fistulization between the RA and the right coronary artery (RCA) causing hemopericardium [22]. Sudden death remains the ultimate devastating presentation of cardiac AS [23]. Hematogenous embolic dissemination of cardiac AS can originate from either the right or the left-side cardiac chambers. Metastatic lesions were reported in lungs [24], liver [25], brain [26], and even within the cervical spine [27].

Physical assessment, electrocardiography, and chest X-ray usually provide limited and non-specific findings. Conventional two-dimentional TTE remains the initial investigational tool used to assess cardiac symptoms. It has its limitations however such as narrow examination windows and operator dependancy. TOE, on the other hand, can provide better access to cardiac structures but it needs special arrangements, a trained cardiologist, and carries some morbidity. Both TTE and TOE offer an excellent diagnostic sensitivities of 93.3% and 96.8% respectively for primary cardiac tumors [28]. Some authors advocate the use of real-time three-dimentional echocardiography for better structural visualization of cardiac AS and their surrounding structures [29].

Computed tomography, especially multi-slice computed tomography (MSCT), is another important radiological tool in the visualization of both primary cardiac AS and their metastatic lesions. Some tumors can be missed however [30]. Some authors reported using a combination: MSCT and positron emission tomography (PET) in order to improve the diagnostic yield and determine the malignant nature of intra-cardiac AS [31]. MRI of the heart has become the reference imaging method for evaluation of suspected cardiac tumors. Mohrs et al reported 100% sensitivity, 100% specificity, 100% positive and negative predictive values for hyper-perfused and for iso-perfused primary cardiac lesions [32]. Tissue biopsy remains the diagnostic gold standard. It must be carried by an experienced interventionalist because of the numerous possible complications (cardiac perforation, arrhythmias, embolic events, etc.).

The battery of therapeutic interventions for primary cardiac tumors keep on growing. At an early stage, radical surgical resection remains essential for improving survival rate of primary cardiac AS. Patients who underwent surgery had a median survival of 12 months whereas those who did not undergo surgery had a median survival of only 1 month [10]. Depending on the location of the tumor within the heart, surgical intervention often requires atrial or ventricular reconstruction [33, 34]. Some specialized centers tried heart transplantation or combined heart and lung transplantation for non-resectable primary cardiac sarcoma [35, 36]. The extremely high recurrence of metastatic disease and the prohibitive costs of these surgeries limit their usefulness, however.

Chemotherapy, with different regimen, is often used for the treatment of sarcomas [2, 20, 37, 38]. The role of radiation therapy has not been established as for malignant cardiac tumors [39]. Finally, some authors propose using the tumor marker CA125 as a good indicator for therapeutic efficacy in the treatment of AS [40].

Despite all therapeutic interventions, primary cardiac ASs have a notoriously grim outcome with a median overall survival ranging from 6 to 14 months [10, 41, 42].

### Conclusions

The coincidental and disastrous occurrence of primary cardiac AS and pregnancy is extremely rare. The physiopathology behind this dreadful combination is not fully understood. Further research into this field is warranted. The clinical presentation can be insidious and challenging. Physicians are encouraged to have a high index of suspicion and vigilance in order to diagnose theses aggressive tumors. Despite urgent multi-faceted therapeutic interventions, AS cordis still has an unrelenting down-spiral course with a dismal outcome.
